# Writing in a Digital World: Self-Correction While Typing in Younger and Older Adults

**DOI:** 10.3390/ijerph121012723

**Published:** 2015-10-13

**Authors:** Yoram M. Kalman, Gitit Kavé, Daniil Umanski

**Affiliations:** 1 The Department of Management and Economics, The Open University, 1 University Road, 43537 Ra’anana, Israel; E-Mails: yoramka@openu.ac.il (Y.M.K.); daniil.umanski@gmail.com (D.U.); 2 The Department of Education and Psychology, The Open University, 1 University Road, 43537 Ra’anana, Israel

**Keywords:** aging, language production, editing, human-computer-interaction

## Abstract

This study examined how younger and older adults approach simple and complex computerized writing tasks. Nineteen younger adults (age range 21–31, mean age 26.1) and 19 older adults (age range 65–83, mean age 72.1) participated in the study. Typing speed, quantitative measures of outcome and process, and self-corrections were recorded. Younger adults spent a lower share of their time on actual typing, and demonstrated more prevalent use of delete keys than did older adults. Within the older group, there was no correlation between the total time spent on the entire task and the number of corrections, but increased typing speed was related to more errors. The results suggest that the approach to the task was different across age groups, either because of age or because of cohort effects. We discuss the interplay of speed and accuracy with regard to digital writing, and its implications for the design of human-computer interactions.

## 1. Introduction

With the introduction of digital devices such as computers and mobile phones, much of our daily communication involves online writing, and this is true also for older adults [1]. Yet, it is unclear how such tasks might be affected by age. It has been found that handwriting speed decreases with age (e.g., [2]), as expected by the general reduction in processing speed on both cognitive and motor tasks that occurs with age [3,4]. It has also been shown that spelling abilities decline with increased age [5,6,7,8]. It is possible that older adults slow down because they correct more errors or that they work more slowly in order to avoid making errors. Previous studies have shown that across different tasks older adults tend to be more cautious even when encouraged to be fast (e.g., [9,10,11]). We set out to examine age differences in digital writing, with a focus on the tradeoff between speed and accuracy.

Most studies of the effects of age on writing speed have looked at handwriting rather than at typing. In fact, when Dixon, Kurzman, and Friesen [12] conducted their seminal study on writing speed in 1993, their participants reported that 87% of their writing was done by hand rather than through typing. This study used copying tasks to record the speed of writing and documented significant age-related decrease in speed, with less pronounced age differences on familiar than on unfamiliar tasks. Later studies that tested both copied and self-generated texts found that speed decreased with age on both types of tasks [13,14]. An analysis of the components of handwriting documented an age-related increase in both on-paper and in-air time, but the ratio between on-paper and in-air time showed the greatest age-related difference, suggesting that writing was less automatic and involved more planning in older age [15]. Furthermore, handwriting speed is related to measures of working memory [16]. Yet, working memory has a more minor effect on handwriting speed, and instead is more important for the cognitively demanding processes involved in writing, such as idea generation, translation of ideas into words, or the formulation of sentences and discourse structures [16].

According to a review of handwriting in old age, older adults report that their writing difficulties include attempts to increase writing speed, spelling mistakes, pen or surface problems, carelessness, lapses in concentration, and external distractions [17]. Despite these reports, this review found no clear evidence of an age-related increase in error rate while writing. On a copying task administered to 80 individuals in four age groups (31–45, 46–60, 61–75, 76+), there was no age-related increase in errors [15]. A study of handwriting in 30 older adults reported that error correction was found in 93% of participants, averaging 3.2 errors per 100 words, yet no age group comparison was conducted [18].

Early research on typing in old age documented little differences in error rates with age, although the focus was primarily on expert typists. In 1984 Salthouse [19] showed that older professional typists were slower on tasks of motor control (e.g., tapping), but demonstrated no decline in typing speed and no increase in errors. Experience either improved performance or helped typists develop compensation mechanisms that led to comparable typing performance across age groups. Salthouse [19] argued that expertise led to more extensive anticipation of impending keystrokes and that this practice served as a compensation mechanism that offset the effects of age. In 1993 Bosman assessed the latency to type a single key or two consecutive keys instead of a continuous text [20]. Skill, but not age, affected speed of typing as well as error rate. A study from 2011 that looked at speed of typing in the general older population (rather than in expert typists), and measured the inter-keystroke interval of login data, showed that the rate of typing one’s login correlated significantly with the rate of performance on a finger tapping task, thus strengthening the assumption that typing of familiar content could reveal one’s motor speed [21]. No analysis of age-related differences was conducted in this study.

The literature on handwriting and typing suggests that speed of performance decreases with age, especially in non-professional typists, but no significant effects of age on error rates have been documented. Yet, older adults report that their spelling abilities decline with age [5,22]. These subjective reports have been generally supported by objective research that focused on spelling of single words [5,6,7,8]. Age-related spelling difficulties are assumed to represent retrieval problems at the process level rather than impairment at the level of orthographic representations, so that detection of misspellings is mostly unaffected by age [6,8,23]. Several factors interact with age-related changes in spelling performance, including individual differences in spelling abilities as well as word frequency [22]. Previous studies have tested production through the ability to correct misspellings [6], through copying of presented words [23], or through writing to dictation of either single words or full sentences [22,24]. In these studies spelling is mostly examined for target stimuli that are difficult to spell, including infrequent words, words with irregular spelling, or homophones, often in contexts that encourage misspellings (e.g., [25]). These contexts might be different from spontaneous writing, especially when writing is done on a computer rather than by hand.

In summary, previous studies suggest that speed of writing decreases with age but that the reduction in speed is offset by typing expertise; that age-related decline in cognitive processes (e.g., working memory) has a minor effect on speed of writing and a stronger effect on higher-order writing; and, that older adults might write less automatically than younger adults. In addition, there is inconsistent evidence concerning error rate in spontaneous writing, despite reports of greater spelling difficulties in old age. It is possible that errors are absent in simple tasks (e.g., copying) but will be found in more complex tasks (e.g., writing a letter). Finally, little is known about the unfolding of the actual production process or the use of editing. Thus, the aim of the current study is to examine age-related differences in the approach to computerized writing with a focus on self-corrections. We compare writing of simple material (listing days of the week and names of months) that involves little planning in terms of content, with more complex self-generation of text (formulating email responses and re-telling the familiar Little Red Riding Hood story). We predict that older adults will type more slowly and will make more errors, and that differences will be especially noticeable on more complex tasks. However, because older adults most likely expect to have more spelling errors, their lower speed might not reflect only the effect of motor slowing but also an attempt to avoid errors and trade speed for accuracy. As age-related differences in editing behavior have not been tested before, we make no specific predictions with regard to the extent of online edits that will be performed by our participants.

## 2. Experimental Section

### 2.1. Participants

A convenience sample of 38 native Hebrew speakers was recruited, half young and half old. The younger group included 19 volunteers (9 women), aged 21–31 (mean = 26.16, *SD* = 3.29), with 12–20 years of education (mean = 14.12, *SD* = 2.40). The older group included 19 community dwelling volunteers (12 women), aged 65–83 (mean = 72.11, *SD* = 5.63), with 10–24 years of education (mean = 14.47, *SD* = 3.27). There was no significant difference in years of education between the two groups, *t* (37) = −0.396, *ns*. To be included in the study, participants had to reply positively when asked if they had any previous experience in using email. All participants reported having no history of learning disorders, psychiatric disturbances, neurological disease, or head trauma, and had normal or corrected-to-normal vision.

The study was conducted in accordance with the Declaration of Helsinki, and the protocol was approved by the Ethics Committee of The Open University. Participants gave their informed consent for inclusion prior to enrollment.

### 2.2. Material and Procedure

Table 1 presents a summary of the study design. We used tasks that recorded simple typing behavior (tasks 1 and 2), and tasks that recorded more complex behavior (tasks 3–5). All instructions appeared on the screen and were available throughout task administration. Each task was followed by a text box in which participants were to type their responses. On the complex tasks participants also saw a note that said that there was no limitation on the length of response, and that the text box would expand if needed.

**Table 1:** Study design.

Task Type	Task Number	Task Content
Simple	Task 1	Days of the week
	Task 2	Months of the year
Complex	Task 3	Response to complaint about cracks
	Task 4	Response to complaint about intersection
	Task 5	Little Red Riding Hood story

#### 2.2.1. Simple Tasks

These tasks were used to record baseline typing of self-generated text. They required no planning in terms of content and involved familiar material with a predetermined order:

Task 1: The instructions were: “Please type the days of the week in their correct order”.

Task 2: The instructions were: “Please type the months of the year in their correct order”.

#### 2.2.2. Complex Tasks

Three tasks were used to record the ability to generate content and translate it into typed material. On tasks 3 and 4 participants were told to “Imagine that you work in the municipality and your job is to respond to residents’ inquiries. Please answer the following two inquiries that were received by email. After each inquiry you will be presented with specific instructions regarding the required response”. The specific instructions were presented one after the other in a numbered list. On task 5 participants were asked to write the Little Red Riding Hood story.

Task 3: The resident’s inquiry was: “Hello, My name is Rebecca Cohen and I live on Herzl Street. There are cracks on the pavement across my house and they endanger pedestrians. Please repair them. Thank you, Rebecca Cohen”. The instructions were to include in the response: (a) an expression of thanks to the resident; (b) an explanation that her inquiry was important to the municipality; (c) reference to the fact that municipal employees will examine the cracks within the next five work days.

Task 4: The resident’s inquiry was: “Hello, My name is Raffi Levy and I live on Ben Gurion Street. Over the last six months there were several accidents on the crosswalk in front of my house. I hear screeching brakes of cars that stop there abruptly several times a day. I ask that you place a traffic light at the intersection. I will appreciate it if you could take care of the problem as soon as possible, Raffi Levy”. The instructions were to include in the response: (a) an expression of thanks to the resident; (b) an explanation that his inquiry was important to the municipality; (c) reference to the fact that municipal employees will arrive to examine the intersection; (d) an explanation that the procedure will first involve placement of a flashing traffic light and if that did not improve the situation, a regular traffic light would then be considered; (e) reference to the fact that the procedure will take time; (f) an explanation that the decision where to place traffic lights is taken together with the police.

Task 5: The instructions were: “Below are five drawings that depict the story of Little Red Riding Hood. Please write the story in your own words. It is important that the story include at least five sentences”. Five colored pictures that were numbered according to the sequence of the original story were presented above the text box.

#### 2.2.3. Task Administration

All tasks were administered in the same order, individually, at the participant’s home or office, and the total time of administration was approximately 30 min. Tasks were administered on a Lenovo T520 laptop (Lenovo Group Ltd., Beijing, China) with a 15.6-inch screen. Participants were asked to type their responses on a standard external keyboard, and they could use an external USB connected mouse. Text boxes into which participants typed their responses resembled a typical word processing file. No text could be imported (e.g., from the experimental instructions), menus were disabled, the automatic spell check and grammar check were deactivated, and there was no access to dictionaries.

The laptop included a key logger that recorded every keystroke at a precision of 1/1000th of a second, so that analyses could track which key was used and when. The key logger was programmed specifically for this experiment, working in the background with participants unaware of its existence. In addition to the logger, we used a commercial screen capture tool called Camtasia (TechSmith Corporation, Okemos, MI, USA) to record all on-screen activity. Finally, a small clock displayed the system’s time throughout the experiment in the format 12:12:35:023. The screen capture tool and the screen clock made it possible to associate the logger data with the actual online writing events that occurred at each point in time, thus documenting not only word typing but also self-corrections made while typing.

### 2.3. Typing Variables

We used the following measures: variables derived from the outcome text; variables derived from the process of typing; variables that documented self-corrections. Algorithms were developed to calculate the variables from the raw typing data. Measures were calculated for each participant and for each task separately and then collapsed into simple or complex task scores.

#### 2.3.1. Outcome Variables

Number of outcome letters: The number of letters in the final text as calculated by the RiTa tokenizer [26]. This tool counts and classifies characters (distinguishing letters from non-letter characters, such as punctuation marks).

Number of outcome errors: Incidences of mistyping and misspelling were counted manually, and included letter omission, letter substitution, letter transposition, or letter addition. Hebrew is mostly written with no vowels but it is possible to mark /i/, /o/, or /u/ with the use of two common letters. The omission and addition of these vowels were generally accepted as correct spelling even if they were non-standard. An unnecessary space was counted as an error if it appeared within a word, but it was not counted as an error if it appeared between words. A missing space between words was counted as an error, but missing spaces were not counted as errors if they appeared before or after punctuation marks. The lack or overuse of punctuation marks was disregarded. Typing the wrong order of days or months or missing a day or a month was considered an error. Missing closed class elements as well as words that were clearly unrelated to the sentence were counted as errors, but poor word choice was ignored (e.g., referring to a fox instead of a wolf on task 5).

#### 2.3.2. Process Variables

Total time: The total time (in seconds) to complete each task was computed from start to finish.

Word time ratio: An algorithm was developed to segment the raw stream of typed characters into words through the detection of likely word boundaries (Space and Enter keys). A series of letters typed between two word boundaries was identified as a word. Once words were identified, the time (in seconds) spent on typing the identified words was summed, and then divided by the total task time. This measure includes time spent on typing letters, time spent on hesitation in the middle of typing a word, as well as deletion and modification before moving to the next word. Word time ratio excludes pausing between words or any editing that occurred after one word was typed and before typing of the next word began.

Time per key: We first counted all keys that were used during typing, including letters, numbers, punctuation marks, parentheses, or any other character, as well as keys that did not produce a visible mark, such as spaces, deletions, or the return key. The average time per key was derived by dividing the total task time (in seconds) by the total number of keys.

#### 2.3.3. Self-Correction Variables

Delete-to-keys ratio: We first counted the number of times the delete or backspace keys were used while typing and then calculated the percentage of these keys out of all keys that were used while typing.

Editing: This variable measured the differences between outcome and process. The calculation was based on the Levenshtein distance between two texts (as described in [27]), which is a measure of the minimal number of single keystrokes needed to edit one text in order to reach a second text. For example, the Levenshtein distance between “kitten” and “sitting” is 3, since it requires two substitutions to transform one into the other (“s” for “k” and “i” for “e”), as well as one insertion (“g” at the end). We compared the distance between the outcome text and the keystrokes used while typing. Raw measures were normalized by dividing the distance count by the length in letters of the longer text. This calculation resulted in a number between 0 and 1, which was then multiplied by 100. A score of 0% indicates that the typing process resulted in the outcome text, with no edits. Higher scores indicate more edits.

## 3. Results

Data from tasks 1 and 2 were collapsed together, serving as baseline, and data from tasks 3–5 were collapsed separately and compared to baseline. A 2 × 2 analysis of variance (ANOVA) was conducted for each variable, with task as a within-subject variable (simple, complex) and age group as a between-subject variable (young, old). Because we ran seven separate analyses on the same data, we used the Bonferroni correction and set our significance level at 0.05/7 = 0.007. Comparisons that did not meet this stringent criterion are marked within the text. In addition to these ANOVAs we also performed an analysis of the correlations among the variables that were derived from the complex tasks, for each age group separately. All analyses were conducted on SPSS.

Table 2 presents raw scores on all variables by type of task and age. As expected, the simple tasks resulted in fewer outcome letters than did the complex tasks, with a significant main effect of task, *F* (1, 36) = 112.90, *p* < 0.007, partial η^2^ = 0.758. The age group difference was not significant, *F* (1, 36) = 3.90, *ns*, and there was no significant interaction between task and age, *F* (1, 36) = 3.70, *ns*. The analysis of errors revealed a significant main effect of task, *F* (1, 36) = 31.13, *p* < 0.007, partial η^2^ = 0.464, with more errors seen on the complex tasks. Older adults had more errors in the outcome than did younger adults, *F* (1, 36) = 11.04, *p* < 0.007, partial η^2^ = 0.235, with younger adults making no errors at all on the simple tasks. The interaction between task and age group was also significant, *F* (1, 36) = 8.17, *p* < 0.007, partial η^2^ = 0.185, as the difference in errors between the two types of tasks was more pronounced in the older group.

**Table 2:** Mean raw scores on simple and complex tasks, by age group.

	Task Type	Young			Old		
		Mean	SD	Range	Mean	SD	Range
No. of outcome letters	Simple	88.42	4.73	75–90	86.11	7.96	73–98
	Complex	849.21	434.29	468–2461	613.58	298.12	264–1422
No. of outcome errors	Simple	-	-	-	0.74	1.33	0–5
	Complex	1.05	1.51	0–6	4.00	3.65	0–14
Total time (in seconds)	Simple	34.34	11.38	17.06–57.98	172.78	125.77	34.04–518.44
	Complex	531.39	433.09	159.68–2191.84	1315.44	600.39	648.33–3309.99
Word time ratio	Simple	0.49	0.17	0–1	0.67	0.23	0–1
	Complex	0.53	0.16	0.31–1	0.68	0.14	0.47–0.93
Time per key (in seconds)	Simple	0.27	0.07	0.17–0.44	1.43	0.94	0.31–3.68
	Complex	0.38	0.11	0.20–0.60	1.70	0.91	0.58–3.88
Delete-to-keys ratio	Simple	0.04	0.04	0–0.11	0.03	0.04	0–0.12
	Complex	0.08	0.04	0.01–0.21	0.05	0.04	0–0.12
Editing (%)	Simple	4.82	4.31	0–14.58	6.37	6.81	0–21.08
	Complex	14.66	10.27	1.91–38.95	8.41	6.72	0.36–22.60

The analysis of total task time yielded the expected effects, with less time spent on typing the simple tasks than on typing the complex tasks, *F* (1, 36) = 106.82, *p* < 0.007, partial η^2^ = 0.748, and younger adults spending less time overall than older adults, *F* (1, 36) = 24.89, *p* < 0.007, partial η^2^ = 0.409. The interaction between task and age group was significant as well, *F* (1, 36) = 16.56, *p* < 0.007, partial η^2^ = 0.315. Younger adults were especially fast on the simpler tasks, completing them in a fifth of the time that it took the older adults, with a smaller difference in magnitude on the complex tasks. When analyzing the time spent on words out of the total task time, only the main effect of age group was significant, *F* (1, 36) = 14.95, *p* < 0.007, partial η^2^ = 0.293. There was no significant main effect of task, *F* (1, 36) = 0.486, *ns*, as well as no significant effect of interaction, *F* (1, 36) = 0.184, *ns*. This analysis shows that older adults spent about 70% of their typing time on words, whereas younger adults spent only about 50% of their time on typing words. The analysis of the time spent on each key revealed a significant main effect of task, *F* (1, 36) = 11.85, *p* < 0.007, partial η^2^ = 0.148, as well as a significant main effect of age group, *F* (1, 36) = 36.56, *p* < 0.007, partial η^2^ = 0.504. There was no significant interaction between task and age groups, *F* (1, 36) = 1.84, *ns*. Thus, all participants typed each key more quickly on the simple tasks than on the complex tasks, and younger adults were faster than were older adults across tasks.

An analysis of the delete-to-keys ratio found a significant effect of task, *F* (1, 36) = 17.45, *p* < 0.007, partial η^2^ = 0.326. The main effect of group was significant as well, *F* (1, 36) = 4.40, *p* < 0.05, partial η^2^ = 0.109, and so was the interaction between task and age group, *F* (1, 36) = 4.66, *p* < 0.05, partial η^2^ = 0.115, although these latter two comparisons did not meet our corrected level of significance. Thus, more delete keys were used on the more complex task, younger adults used a greater proportion of delete keys than did the older adults, and the difference in delete use across tasks was greater within the younger group than it was within the older group. The analysis that looked at editing found a main effect of task, *F* (1, 36) = 14.21, *p* < 0.007, partial η^2^ = 0.283, as well as an interaction between task and age group, *F* (1, 36) = 6.14, *p* < 0.05, partial η^2^ = 0.146, although the effect of interaction did not meet our corrected level of significance. The main effect of age group was not significant, *F* (1, 36) = 1.73, *ns*. As can be seen in Table 2, the interaction reflects the fact that younger adults made few edits on the simple task and twice as many edits on the complex tasks, whereas older adults made a similar percentage of edits across tasks.

Finally, we calculated the correlations between process variables and self-correction variables on the complex tasks for each group separately. These correlations are presented in Table 3. The results show that younger adults who took more time to complete the tasks used more delete keys relative to the other keys and edited more, so that the correlations between total time and these measures were positive and high within the young group. There were no such correlations within the older group. In contrast, the rate of typing, as measured by the time to type each key, significantly correlated with the percentage of editing within the old group but not within the young group. This finding indicates that older adults who typed fast also edited more, whereas younger adults edited their texts regardless of their typing rate.

**Table 3:** Correlations between process variables and self-correction variables for the complex tasks, by age group.

Variable	Age Group	Total Time	Word Time Ratio	Time per Key	Delete-to-Keys Ratio
Word time ratio	Young	0.080			
	Old	0.370			
Time per key	Young	0.092	0.768 **		
	Old	0.344	0.871 **		
Delete-to-keys ratio	Young	0.745 **	0.099	−0.002	
	Old	0.049	−0.207	−0.264	
Editing	Young	0.753 **	0.036	−0.014	0.861 **
	Old	−0.016	−0.494 *	−0.552 *	0.756 **

Notes: ** α = 0.001; * α = 0.05.

## 4. Discussion

In this study we examined how younger and older adults approach a writing task that was completed on a computer. We looked at outcome quantity, error rate, timing variables, and indications of self-corrections. Our results show that younger adults appear to type more than do older adults on the more complex tasks, but this difference did not reach significance, potentially because of the variance in performance as well as the size of our sample. Despite the fact that older adults tended to type less, they took significantly more time to complete the task relative to the younger adults. Older adults had more time to correct their errors, but they used this time differently. Thus, their final texts included more errors than did the final texts produced by the younger participants, and they also made fewer corrections while typing. When examining the time that was dedicated to writing as opposed to pausing, planning, or editing, we found that older adults spent about 70% of their time on actual typing, whereas younger adults spent only about half of their time typing. As expected, the speed of typing, as calculated by the time to type each key, was much higher in the younger group. In addition, younger adults edited their texts more extensively on the complex tasks than on the simple tasks, whereas older adults showed no such difference. The correlation analyses further demonstrated that the overall time that younger adults spent on the task was associated with greater deleting and editing, with no comparable associations documented in the older group.

Our data reflect differences in task approach that could be the result of age or cohort, or both. It is possible that older adults find the actual typing more difficult because of a decline in motor skills, timing, or sequencing [28]. A decrease in speed of typing or a more general decrease in speed of processing [3,4,19] could have also affected the time that it took to plan one’s message. In addition, age-related reduction in working memory capacity could have affected typing behavior, although working memory may have a greater effect on higher-order text-generation than on the lower-level transcription process, as measured by speed and spelling [16]. Most importantly, older adults could slow down to avoid errors, as previous studies have shown that difficulties in spelling increase with age [5,6]. Even though we found that older adults had more errors in the outcome text than did younger adults, older participants could still be more cautious while typing so as to avoid misspellings and other errors. The fact that they make fewer self-corrections while typing relative to the younger participants might indicate that they use a different approach to the task. Hence, the older writer might take into account the combined effects of age on one’s typing, being partially aware of decreased speed, limited working memory resources, and increased difficulties with spelling, and thus slowing down to plan before executing any keystroke.

Alternatively, the observed age differences may also reflect cohort effects. As younger adults are more experienced with computers or other electronic devices (e.g., mobile phones, tablets) than are older adults, they might be more used to writing and rewriting. Indeed, previous studies found age-related differences in word-processing performance even among computer novices [29], suggesting that there are cohort differences in these skills. Differences could result, for example, from the pattern of keyboard-screen gaze time allocation [30]. In addition, over their life course older adults have become accustomed to writing primarily by hand (or possibly typing on a typewriter). Extensive editing of handwriting takes a more significant toll than does extensive editing on a computer. Younger adults might have become used to the fact that writing involves many deletions and changes. Thus, they trade accuracy for speed when first generating their text, knowing that they will correct themselves later, whereas older adults might be more inclined to get it right the first time. Our results show that younger adults spent only about half their time on word typing, dedicating the rest of their time to pausing, reviewing, and editing their text. In contrast, older adults spent about 70% of their time on word writing and editing, so that even if they corrected themselves, more of their edits were likely done at the word level.

Since we used a cross-sectional design, we cannot determine whether our results reflect age effects, cohort effects, or the combination of both types of effects. Future research should follow individuals longitudinally to tease these effects apart. Had we asked our participants about their familiarity with computers or the time that they spend writing on electronic devices, we could have incorporated this information into the analyses to better understand the importance of previous experience. However, since typing on personal electronic devices has become common only in recent decades, we could not use statistical means to control for the differential ratio between handwriting and typing over one’s life that exists across cohorts. As research on editing and self-corrections while typing on digital devices is not very extensive, we had to develop novel measures and algorithms to quantify these activities. These measures must be further investigated along with other aspects of computerized writing, such as the intra-individual variance in performance that might occur within or between tasks. We note that order effects could have also contributed to the documented age differences, as the complex tasks were always presented after the simple tasks. Even though the entire administration was relatively short, fatigue could have had a greater impact on older adults, and future research should address this issue.

## 5. Conclusions

The current study extends our understanding of the process of writing by identifying individual differences not only in the quality of the final text but also in online typing behavior and rate of editing. Since language can reflect neurological injury and even predict subsequent cognitive decline [31,32], our findings enrich the pool of variables that might be used as human-computer-interaction (HCI) markers for such decline. A better understanding of individual differences in online writing can also contribute to HCI design, especially in the context of personalized adaptive accessibility [33], an approach that emphasizes the fit between users’ abilities and the computer interface.
